# Liverome: a curated database of liver cancer-related gene signatures with self-contained context information

**DOI:** 10.1186/1471-2164-12-S3-S3

**Published:** 2011-11-30

**Authors:** Langho Lee, Kai Wang, Gang Li, Zhi Xie, Yuli Wang, Jiangchun Xu, Shaoxian Sun, David Pocalyko, Jong Bhak, Chulhong Kim, Kee-Ho Lee, Ye Jin Jang, Young Il Yeom, Hyang-Sook Yoo, Seungwoo Hwang

**Affiliations:** 1 Korean Bioinformation Center, Korea Research Institute of Bioscience and Biotechnology, Daejeon, Korea; 2 Pfizer Global Research and Development, San Diego, CA, USA; 3 Theragen BiO Institute, Suwon, Korea; 4 Laboratory of Radiation Molecular Cancer, Korea Institute of Radiological and Medical Sciences, Seoul, Korea; 5 Medical Genomics Research Center, Korea Research Institute of Bioscience and Biotechnology, Daejeon, Korea; 6 Daejeon-KRIBB-FHCRC Research Cooperation Center, Korea Research Institute of Bioscience and Biotechnology, Daejeon, Korea

## Abstract

**Background:**

Hepatocellular carcinoma (HCC) is the fifth most common cancer worldwide. A number of molecular profiling studies have investigated the changes in gene and protein expression that are associated with various clinicopathological characteristics of HCC and generated a wealth of scattered information, usually in the form of gene signature tables. A database of the published HCC gene signatures would be useful to liver cancer researchers seeking to retrieve existing differential expression information on a candidate gene and to make comparisons between signatures for prioritization of common genes. A challenge in constructing such database is that a direct import of the signatures as appeared in articles would lead to a loss or ambiguity of their context information that is essential for a correct biological interpretation of a gene’s expression change. This challenge arises because designation of compared sample groups is most often abbreviated, *ad hoc*, or even missing from published signature tables. Without manual curation, the context information becomes lost, leading to uninformative database contents. Although several databases of gene signatures are available, none of them contains informative form of signatures nor shows comprehensive coverage on liver cancer. Thus we constructed Liverome, a curated database of liver cancer-related gene signatures with self-contained context information.

**Description:**

Liverome’s data coverage is more than three times larger than any other signature database, consisting of 143 signatures taken from 98 HCC studies, mostly microarray and proteome, and involving 6,927 genes. The signatures were post-processed into an informative and uniform representation and annotated with an itemized summary so that all context information is unambiguously self-contained within the database. The signatures were further informatively named and meaningfully organized according to ten functional categories for guided browsing. Its web interface enables a straightforward retrieval of known differential expression information on a query gene and a comparison of signatures to prioritize common genes. The utility of Liverome-collected data is shown by case studies in which useful biological insights on HCC are produced.

**Conclusion:**

Liverome database provides a comprehensive collection of well-curated HCC gene signatures and straightforward interfaces for gene search and signature comparison as well. Liverome is available at http://liverome.kobic.re.kr.

## Background

Hepatocellular carcinoma (HCC) is the fifth most common and the third most deadly cancer worldwide, accounting for 600,000 deaths annually [1]. Clinical observations have established that most HCC patients have underlying chronic liver diseases; 70-80% of HCC patients suffer from liver cirrhosis caused by chronic infection of hepatitis B virus or hepatitis C virus, excessive alcohol consumption, or certain metabolic disorders. In addition, male gender and exposure to environmental toxins such as aflatoxin increase the risk of developing HCC. Further, recent genetic and genomic studies have pointed to germline or somatic DNA alterations and specific molecular pathways for the initiation and progression of HCC. Currently, only one third of newly diagnosed HCC patients are eligible for potential curative therapies, mainly due to a lack of early detection technology and limited choices of effective therapeutic agents [2]. Early accurate diagnosis and effective treatment of HCC require a deep understanding of how it occurs at a molecular and cellular level.

Advances in high-throughput technologies in transcriptomics and proteomics have changed the way in which HCC is studied and, potentially, diagnosed and classified in clinical practice. During the last decade, a number of molecular profiling studies have investigated changes in gene and protein expression that are induced by HCC. While early studies had focused on the comparison of expression profiles between tumorous and non-tumorous liver, later studies have more fully explored the molecular pathogenesis of HCC by associating expression changes with various clinicopathological characteristics of HCC such as etiology, prognosis, metastasis, and HCC subgroups.

The result from the molecular profiling studies most often comes in the form of a list of genes, also called a gene signature, which is reported in research articles as a table in the main text or a supplementary table. The gene signature typically consists of gene identifiers and numerical ranking information such as fold change, *p*-value, and other relevant statistics that describe the nature and the strength of association between the gene and the phenotype under study. Each of the gene signatures derived from profiling studies on HCC gives a distinct molecular portrait of a specific aspect of HCC under a specific sample cohort. Collective information that arises from a wealth of diverse gene signatures is expected to contribute significantly to a comprehensive understanding of molecular events underlying HCC and to the prioritization of candidate target genes for diagnosis and treatment.

Specifically, the collection of published gene signatures can be useful to liver cancer researchers in two most common situations. In one situation, a researcher may hypothesize that a particular gene is implicated in HCC and seek published evidences supporting the hypothesis. For this purpose, the candidate gene can be searched on the gene signature collection to retrieve all available differential expression evidences. If the search shows compelling evidences, the candidate gene can be further examined in depth by experimental means. In the other situation, a researcher might have performed a molecular profiling study to derive a gene signature associated with a particular phenotype under the study, for example, survival of HCC patients. Then, the derived signature can be compared with survival-associated signatures in the collection to identify common genes. Such common genes can be considered as robust marker genes that are reproducible across independent sample cohorts, and their identification can help prioritize the user-derived signature for a further in-depth validation study. To effectively serve as the gene search and signature comparison resource for liver cancer research community, we argue that a gene signature database needs to be constructed with the following considerations on web interface and database content, all of which have not been addressed by other gene signature databases currently available, such as CCancer [3], dbDEPC [4], EHCO [5], and GeneSigDB [6].

With respect to the web interface, the first aspect to consider is that the gene signature collection should be linked to both a gene search interface that retrieves differential expression information of query gene as well as a signature comparison interface that compares user-selected signatures to identify common genes for prioritization. Second, to facilitate signature comparison, the collected signatures need to be meaningfully organized for guided browsing according to their functional category, such as etiology, survival, recurrence, and differentiation. Third, it should be beneficial to give each signature an informative name for a quick recognition and easy browsing. Due to the diversity of HCC gene signatures, a cluttered collection without the meaningful organization and the informative naming would make it difficult to browse through the voluminous gene signature collection.

With respect to the database content, the first aspect to consider is that the gene signature collection should be comprehensive and up-to-date, covering most, if not all, published HCC gene signatures that associate expression changes with various characteristics of HCC. Second aspect worth considering is to present numerical ranking value in a uniform representation. Published gene signature tables present fold change values in various formats, such as ratio, log ratio, fold change with a direction tag, and a pair of intensity values. Presenting them in a uniform format will make it more intuitive to recognize similar data and make comparisons across signatures. Third, and most importantly, context information of gene signatures needs to be curated to the extent that all information is unambiguously self-contained within the database. With the availability of self-contained context information, database users would not have to refer to source articles from which the signatures were reported. The context information of a gene signature comes in two types: (i) summary of experiment that produced the gene signature, and (ii) explicit designation of compared sample groups associated with numerical ranking value (for example, fold change in poorly-differentiated samples compared to well-differentiated samples). The summary of experiment would be helpful for database users to quickly recognize detailed characteristics of gene signatures and it should document platform, clinicopathological sample characteristics, sample size, data analysis method, and others. More crucial piece of information is the explicit designation of compared sample groups associated with numerical ranking value, which is utmost essential for a correct biological interpretation of a gene’s expression change.

**Figure 1:**
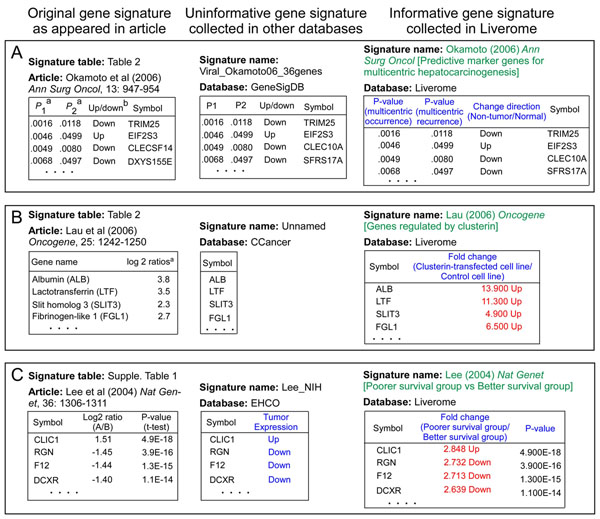
**Examples on how gene signatures appear in articles, in other databases, and in Liverome.** The left, middle, and right columns show gene signatures as appeared in articles, in other gene signature databases, and in Liverome, respectively. Column titles for numerical ranking information were abbreviated or *ad hoc* in the original gene signature tables (left column). Gene signatures extracted by other databases are uninformative (middle column): (A) direct import of original table causes an ambiguity as to what P1 and P2 means and to the context of the observed expression changes (Down and Up), (B) importing only the gene identifiers causes a complete loss of differential expression information, and (C) importing only the change direction causes a loss of numerical information as well as an ambiguity as to the context of the observed expression changes. Shown in blue highlights how the information from the original signature tables became transformed in the databases. Liverome derives the most informative form of gene signatures through manual curation to construct self-contained database content (right column). In addition, for an easier recognition, fold change values were uniformly formatted (shown in red) and signatures were informatively named (shown in green) in Liverome.

Nevertheless, the explicit designation of compared sample groups is most often ignored by other gene signature databases since it can only be prepared through additional manual curation effort. In most gene signature tables appeared in research articles, sample group names are either abbreviated, *ad hoc*, or even missing from the table column title. Instead, the sample groups are usually designated in full in the source article’s table legend or main text. The abbreviated, *ad hoc*, or missing table column title poses no problem with respect to the article itself, but becomes problematic upon incorporating into database. Since other gene signature databases merely extract gene signatures as they appear in the published tables without further manual curation, the sample group designation becomes ambiguous or even lost from the collected gene signatures, leading to an uninformative form of gene signatures. In Figure 1, we show examples of published gene signature tables, uninformative form of gene signatures collected in other databases, and informative form of gene signatures that we derived through manual curation. A database containing the uninformative gene signatures would merely produce a meaningless search result, as illustrated later in the “Comparison to other resources” section. While it would not be impossible for database users to obtain the information on sample group designation from source articles, doing so would be very inconvenient and greatly undermine the practical utility of the database.

**Figure 2:**
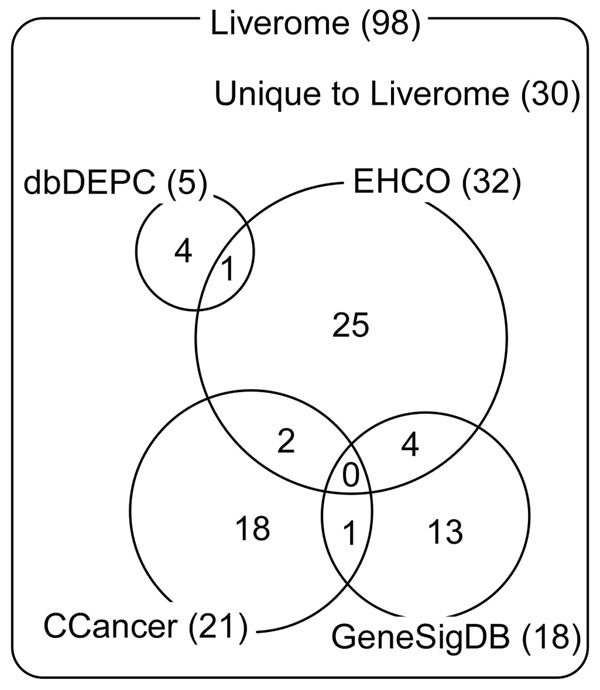
**Comparison of HCC data coverage between Liverome and four other signature databases.** For each of the databases, the number of collected HCC-related articles from which gene signatures were extracted is indicated. The four other databases show minimal overlaps with each other. Liverome’s collection is more than three times larger than any other database. One third of its data (thirty articles) is unique to Liverome. Only the articles that met our collection criteria were counted, as described in the “Construction and content” section.

To address all these unmet needs toward a straightforward gene search and signature comparison resource for liver cancer research community, we constructed Liverome, a curated database of liver cancer-related gene signatures with self-contained context information. Here, the self-contained context information includes both the summary of experiment that produced the signature and the explicit designation of compared sample groups associated with numerical ranking value. Its collection consists of 143 signatures compiled from 98 HCC-related profiling studies, mostly microarray and proteomics, and involves 6,927 genes. The size of our HCC signature collection is more than three times larger than any other signature database (Figure 2). The collection was manually post-processed, annotated, and systematically organized in order to provide informative and self-contained database contents. The database can be accessed through two web interfaces: (i) a gene search interface that retrieves self-contained differential expression information on query gene, consisting of numerical ranking value in uniform representation, explicit designation of compared sample groups, and summary of experiment, and (ii) a gene signature comparison interface that facilitates guided browsing of signature collection and comparison of user-selected signatures to identify common genes, ordered by occurrence frequency. The uniqueness of Liverome in terms of database size, database content, and web interface, are summarized later in the “Comparison to other resources” section. Liverome is most useful to retrieve well-curated supporting evidences from published molecular profiling studies in HCC on a user-specified candidate gene or gene signature. In profiling studies, authors usually select a handful of genes for validation or discussion in the article without paying much attention to the rest of the genes in their signature. With Liverome, underused information that is buried within the previously reported HCC gene signatures can be brought back to the surface. Liverome is available at http://liverome.kobic.re.kr.

## Construction and content

### Collecting and extracting gene signatures

We collected 98 HCC-related profiling studies, consisting of 83 transcriptomic studies, 12 proteomic studies, and three other genome scale studies. We collected only those articles that met our collection criteria; (i) articles should report novel experimental findings (that is, we excluded review articles), (ii) the experiment should be done on HCC patients or human HCC cell lines (that is, we excluded studies done on mouse models), and (iii) the article should report gene signature tables containing enough genes (which was set arbitrarily to eight or more genes). From the main text and supplementary material of collected articles, we manually extracted gene signatures that are reported as tables (or as heatmap figures in a few cases), totalling 143 gene signatures listed in Table S1 (Additional File 1). Statistics of the gene signatures are further summarized in Table S2 (Additional File 1) with respect to ten functional categories: (1) tumor versus non-tumorous liver comparison, (2) survival/recurrence, (3) cirrhosis/dysplasia, (4) etiology, (5) differentiation, (6) invasion/metastasis, (7) genomic alterations, (8) modulation by a gene/protein factor, (9) HCC subgroups, and (10) other nature.

### Post-processing the extracted signatures into uniformly formatted, informative, and self-contained form

Once the gene signature tables were extracted, we manually post-processed them to convert into a uniformly formatted, informative, and self-contained form. The conversion was made under the following four guiding principles. First, fold change values are presented in non-logarithmic ratio and non-ratio scale along with a direction tag. For example, “7.000 Down” was uniformly used to represent the reported equivalent values such as a ratio value of 0.1428, a log2 ratio value of -2.8073, a text value of ↓7.0, or a pair of intensity values of 50 and 350. Second, the column titles for fold change should explicitly designate case and control sample groups, such as tumor/normal, poor survival group/good survival group, and invasive tumor/non-invasive tumor, in which the case group is designated in the numerator and the control group in the denominator. Third, columns that give inessential information, such as the confidence interval of a test statistic, may be omitted for a succinct representation, or columns that do not exist in the original tables may be added when necessary; an example being a direction tag (up or down). Fourth, a gene signature table may be split into several signatures or several tables may be combined into one signature when appropriate. All the published gene signature tables needed to be post-processed to some extent, and attention to detail was paid throughout the post-processing to give the most informative database content, which was made possible only after first reading and understanding the source articles. The post-processing of gene signatures is a unique feature of Liverome’s database content.

In addition to the numerical ranking information, gene or protein identifiers were extracted and mapped to the most recent EntrezGene IDs using mapping files obtained from NCBI. In the absence of definite identifiers, for example when only the gene names were provided in the published signature tables, corresponding gene identifiers were acquired manually. We excluded entries that cannot be reliably mapped to EntrezGene, such as ambiguous gene names or retired UniGene IDs without further traceable information.

### Annotating gene signatures: informative naming and self-contained documenting

**Figure 3:**
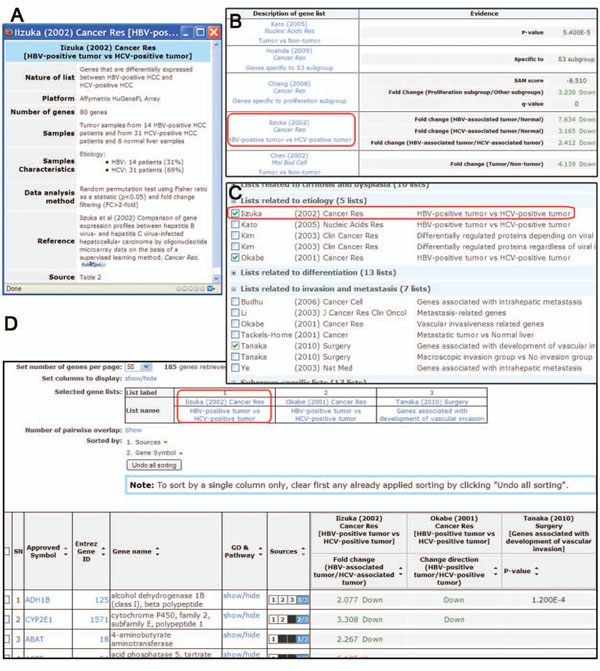
**The web interface of Liverome.** (A) Itemized summary of gene signature, which appears as a pop-up window upon clicking on the name of the signature, as indicated by red rounded rectangles in the figure. (B) Result from gene search interface which reports the informatively named gene signature hits in which the queried gene is found (left column), along with numerical ranking information and designation of compared groups (right column). (C) The browse and comparison interface in which three signatures are marked. (D) Result from the browse and comparison interface. Several display controls are provided at the top of the screen. The table below provides a sorted view of all the genes that are found in the selected signatures. The sequence of the sorting applied to the table is shown on the right of the “Sorted by” control. This table was made compact by using the “Set columns to display” control. See the user guide on the web site for more information.

Subsequent to the post-processing of the extracted signatures, we further annotated them to produce informative signature name and itemized summary. The annotation effort was made in order to help database users recognize the nature of gene signatures without having to read the source articles. First, since an *ad hoc* naming of gene signatures (for example, “Viral_Okamoto06_36genes” shown in the middle of Figure 1A) would significantly hinder the quick recognition, we gave each gene signature an informative name of the form “Author (Year) Journal [Short name]” (for example, “Okamoto (2006) Ann Surg Oncol [Predictive marker genes for multicentric hepatocarcinogenesis]” shown in the right of Figure 1A). For gene signatures obtained from a two-class comparison, the short name generally takes the form of “case vs. control”, such as recurrence group vs. recurrence-free group, tumor vs. non-tumor, and tumor vs. normal liver. As shown in the latter two examples, we made a distinction between non-tumor (adjacent non-tumorous normal tissue) and normal liver (normal tissue from a tumor-free subject) since the two types of control samples have different characteristics [7]. For gene signatures obtained from other types of analyses, either the names used in the source articles or the names that we appropriately designated were used. Second, for each gene signature, we read its source article and prepared a detailed yet concise itemized summary documenting basic nature of the signature, platform, signature length, sample size, clinicopathological sample characteristics, data analysis method, and reference information. The gene signature summary page appears as a pop-up window upon clicking on the name of the gene signature throughout the web site (Figure 3A). The annotation of gene signatures is also a unique feature of Liverome’s data content.

## Utility and discussion

### Web interface

Liverome database is implemented in MySQL, and its web interface is written in PHP and JavaScript under Apache web server running on a Linux system. Liverome provides two types of interface: (i) search interface for a gene, and (ii) browse and comparison interface for gene signatures. The result from the gene search interface is the informatively named gene signature hits in which the queried gene is found, along with numerical ranking information in uniform representation and explicit designation of compared sample groups (Figure 3B). On the browse and comparison interface, gene signatures are organized for guided browsing into ten groups according to their functional categories (Figure 3C). This interface is to be used when one or more collected signatures need to be retrieved and compared, or when users want to compare their signature with collected ones. In all cases, users need to select the desired signatures by marking their checkboxes. Comparing a user-provided signature with all the collected signatures is equivalent to performing a batch query.

The selected signatures are then passed to the next screen in which a combined gene signature table appears (Figure 3D), along with three additional menus in the left: export data, pathway grouping, and GO (Gene Ontology) grouping. The grouping is a simple version of enrichment analysis, in which all the present KEGG pathway or GO terms are reported without applying a *p*-value cutoff. For an advanced enrichment analysis, users can export the accessed gene signature to an Excel file for the use of a third party tool. Several display controls are also provided above the gene signature table, such as the one that allows viewing the number of pairwise overlapping genes between signatures [6,8]. The most important control is “sorted by”. The order of genes in the table can be sorted by multiple columns in a sequential manner (that is, a spreadsheet-like sorting) by clicking the upward or downward arrows in the header row; the “sorted by” field shows the sorting sequence. The default ordering is set in a way that is the most reasonable under the given context, such as a descending sort on occurrence frequency followed by a sort on the gene symbol when multiple signatures are retrieved. The default ordering can be cleared by clicking “undo all sorting” and re-ordered by users. The sorting functionality should be useful to sort out the genes that are supported by multiple evidences or a particular set of evidence. An exercise on sorting is shown in the user guide on the web site.

### Comparison to other resources

There are several recently constructed databases that provide an access to published signatures. One is specific to HCC (EHCO [5]), thus the most directly comparable to Liverome, while the other three are broadly scoped encompassing all types of cancer (dbDEPC [4]) and some additional phenotypes as well (CCancer [3] and GeneSigDB [6]). Each of these resources, including ours, is aimed at its own specific utility, serves particular target user group, and has both strengths and limitations. Thus, we compare them with respect to Liverome’s main utility as a gene search and signature comparison resource for liver cancer research community (Table 1), while acknowledging the benefits offered by each of the resources as well.

**Table 1:** Comparison of Liverome with other related tools.

	Liverome	EHCO	dbDEPC	CCancer	GeneSigDB
**Data coverage**					
					
Coverage of phenotype	HCC only	HCC only	15 cancers	Half the data are on cancer	Mostly cancer and stem cell
Coverage of HCC-specific data (signatures // articles)	143 // 98	12 // 32	6 // 5	25 // 21	34 // 18
Overall data coverage	Same as above	Same as above	65 // 48	3369 // 2644	2142 // 973
Covers both transcriptomics and proteomics studies	**Yes**	**Yes**	No (proteomics only)	**Yes**	**Yes**
**Data content**					
					
Explicit designation of compared sample groups	**Yes**	No	**Yes**	No	No
Contains numerical ranking information	**Yes**	No (change direction only)	**Yes** (fold change only)	No	**Yes**
Uniform representation of numerical ranking values	**Yes** (unique to Liverome)	No	No	No	No
Informative naming of signatures	**Yes** (unique to Liverome)	No	No	No	No
Summary of experiment	**Yes** (unique to Liverome)	No	No	No	No
**Web interface**					
					
Signature comparison tool	Yes	No	**Yes**	**Yes**	**Yes**
Gene search tool	**Yes**	**Yes**	**Yes**	No	**Yes**
Functional categorization of signatures for guided browsing	**Yes** (unique to Liverome)	No	No	No	No
Spreadsheet-like sorting utility for prioritization	**Yes** (unique to Liverome)	No	No	No	No

Liverome is compared to four other gene signature databases with respect to Liverome’s main utility as a gene search and signature comparison resource for liver cancer research community. Liverome achieves the largest coverage of HCC signatures and the most informative data content at the same time. Its web interface is designed to facilitate the retrieval of the informative data content, the guided browsing of signatures, and their comparison for occurrence-based prioritization.

With respect to data coverage, Liverome’s collection of 143 HCC-related signatures from 98 articles represents a data coverage of more than four times larger (signature-wisely) and three times larger (article-wisely) than any other resource. As seen in the Venn diagram of Figure 2, two thirds of Liverome’s collection (68 articles) represents a consolidated data coverage of all other resources while one third (30 articles) is unique to Liverome. Despite the broad scope and overall large coverage attained in CCancer and GeneSigDB, their HCC-specific coverage is merely less than one quarter of Liverome, which points to the need of a gene signature database dedicated to HCC.

**Figure 4:**
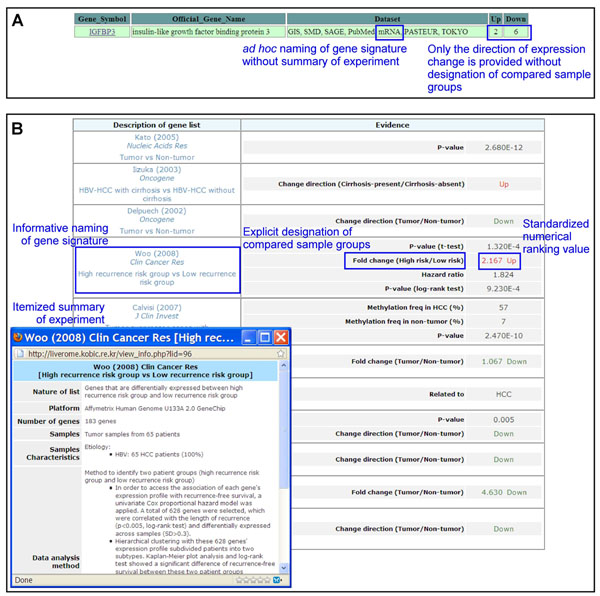
**A gene search result is informative only in the presence of informative data content.** (A) A search for *IGFBP3* gene on EHCO database produces an uninformative result. To decipher the retrieved result, users need to read the source article named “mRNA” to figure out whether the observed expression change of the gene was up-regulation or down-regulation under that dataset as well as the compared sample groups. (B) A search for *IGFBP3* gene on Liverome produces an informative result; all information is readily readable and self-contained in one screen.

With respect to data content, the benefits offered by Liverome’s manual curation are noticeable. Compared sample groups are explicitly designated only in Liverome and one other database (dbDEPC). Three aspects of producing informative content are not addressed by any other database. In particular, EHCO and CCancer did not curate their collected data at all, lacking all five aspects of producing informative data content. In the absence of informative data content, a gene search on the database would not be informative either; the search result would merely indicate the gene signature hits in which the query is found (Figure 4A). Thus, to go beyond recognizing the query gene has some HCC-related evidence, database users would need to figure out the numerical ranking value and the context in which the query gene is associated with HCC by reading source articles. While doing so would not be impossible, it would be very inconvenient and greatly undermine the practical utility of the database. In contrast, a gene search result on Liverome is informative; all information retrieved from the gene search is readily readable and self-contained in one screen (Figure 4B).

With respect to web interface, features unique to Liverome are functional categorization of gene signatures for guided browsing and spreadsheet-like sorting utility for occurrence-based prioritization. Liverome also has a distinctive implementation for signature comparison and gene search interfaces, which are different from corresponding interfaces implemented in other resources. The signature comparison interface of Liverome allows users to select a subset of comparable signatures of similar nature (for example, survival-associated gene signatures), whereas those of other resources do not. The gene search interface of Liverome is designed to produce a search result screen in which all information is readily readable and self-contained in one screen (Figure 4B). Obtaining such information from other resources requires reading the source articles and/or following multiple hyperlinks.

Although Liverome is superior to other resources with respect to its own utility as a gene search and signature comparison tool for liver cancer research community, the other resources also offer benefits not addressed by Liverome. CCancer enables identification of frequently co-reported gene pairs, computes signature overlap *p*-values, and provides by far the largest overall data coverage achieved through an automatic extraction of gene identifiers at the expense of missing numerical ranking information. GeneSigDB computes signature overlap *p*-values, maintains a trace of identifier mapping between the original and the re-mapped signatures, and provides a large overall data coverage even with acquisition of both gene identifiers and accompanying numerical ranking information. dbDEPC provides an explicit designation of the compared sample groups and the largest coverage of cancer-related protein signatures. All three generic resources can be a useful tool for global cross-comparison of heterogeneous cancer types. As for EHCO, the most directly comparable HCC-specific resource, we did not find any major comparative benefits that it offers. It does provide, however, a list of HCC-related genes derived through text mining, which was imported to our database as one of the collected signatures. In addition, they reported in the article an interesting post-construction analysis of protein-protein interaction network using EHCO-collected data.

To summarize, our collection of HCC-related gene signatures is the most comprehensive. Furthermore, we generated the most informative and self-contained database contents through manual post-processing and annotation, which had not been done in other databases. Our web interfaces are the most straightforward for the retrieval of well-curated differential expression information on a query gene, the guided browsing of signature collection, and the comparison of signatures for occurrence-based prioritization of common genes. In the future, we will continuously expand Liverome’s signature collection once a year by incorporating newly published HCC gene signatures. Next we demonstrate the utility of Liverome by presenting several examples in which useful biological insights on HCC are produced.

### Case study 1: genes that are frequently reported among the gene signature collection in Liverome

With a database like Liverome, one natural question that can be raised is which genes are most frequently reported among the various collected gene signatures. To answer this question, we marked all 143 gene signatures from the browse and comparison interface and sorted the genes by occurrence frequency. Out of all 6,927 genes, about half of them occur in only one signature while a few genes occur frequently (Figure S1, Additional File 2). Top 22 most frequent genes occurring in 12 or more signatures are listed in Table S3 (Additional File 1).

Glypican 3 (*GPC3*) was identified as the third most frequently reported gene, observed in 17 gene signatures. *GPC3* is a heparan sulfate proteoglycan that is attached to the cell surface by a glycosylphosphatidylinositol anchor. Previous studies have pointed to its connection to HCC. *GPC3* is highly expressed in HCC [9], stimulates the growth of HCC cells in vitro and in vivo by activating the canonical Wnt signaling [9], and modulates cell proliferation through fibroblast growth factor 2 and bone morphogenetic protein 7 signaling [10]. To confirm that the reported up-regulation of *GPC3* in HCC is a frequent event and is captured by microarray studies across various platforms, we searched for *GPC3* in Liverome. From the 17 gene signature hits that contain *GPC3*, we could easily confirm that the up-regulation of *GPC3* is consistently supported by eight signatures derived from comparison of tumor with non-tumor or normal liver, along with large fold change values (Figure S2, Additional File 2). This collective information further supports the previously suggested usefulness of *GPC3* as a potential diagnostic marker for HCC and a target for antibody-based therapeutic intervention of HCC [11].

In addition, two genes that encode enzymes in the one-carbon metabolism pathway, namely betaine-homocysteine S-methyltransferase (*BHMT*) and methylenetetrahydrofolate dehydrogenase 1 (*MTHFD1*), were identified as the fourth and seventh most frequently reported genes, observed in 16 and 13 signatures, respectively. It is known that epigenetic alterations, such as global hypomethylation, regional hypermethylation of tumor suppressor genes, and histone modification that alters chromatin structure, are involved in HCC. One-carbon metabolism, with S-adenosylmethionine (SAM) as the principal biological methyl donor, is required for the epigenetic events. Both *BHMT* and *MTHFD1* are involved in the synthesis and regulation of SAM. Several lines of evidence have suggested that one-carbon metabolism plays an important role in HCC. First, folate/methyl-deficient rat models have shown that sufficient supply of SAM can prevent HCC development whereas chronic depletion of SAM can lead to HCC [12]. These models also reported an altered expression of epigenetic enzymes such as DNA methyltransferases (DNMTs) and methyl CpG binding proteins (MBDs) [13]. The aberrant regulation of DNMTs and MBDs have been observed in HCC patients and are suggested to be involved in silencing of tumor suppressor genes leading to uncontrolled cell growth and activation of metastasis genes, respectively [14]. Second, it was reported that administration of SAM in rats reduced the expression of c-myc, Hras, and Kras oncogenes in proliferating liver cells and tumor nodules [15]. Third, by searching for *BHMT* and *MTHFD1* in Liverome, we could easily see that down-regulation of the two genes is prevalent in proliferative and poorly differentiated HCCs (Figures S3 and S4, Additional File 2). These evidences might shed light on the role of one-carbon metabolism genes in HCC progression, possibly through epigenetic regulation.

### Case study 2: genes with somatic mutation in HCC

Next, we sought to retrieve supporting information on genes with somatic mutation in HCC using Liverome. All cancers arise from acquisition of a series of fixed DNA sequence abnormalities, many of which ultimately confer a growth advantage to the cells in which they have occurred. COSMIC (Catalogue of Somatic Mutations in Cancer) database [16] provides the somatic mutation information but not the gene expression information. In order to collect comprehensive data on genes with somatic mutation in HCC, we first identified frequently mutated genes in HCC from COSMIC with the following three criteria: primary tumor samples only, a minimum of 5% somatic mutation frequency, and a minimum of ten samples screened. There were eleven such genes. Upon searching for those genes on Liverome, differential expression information of eight of the genes (*CSF1R*, *CTNNB1*, *CDKN2A*, *RB1*, *HNF1A*, *KRAS*, *MET*, and *PTEN*) was retrieved (Table S4, Additional File 1), which may provide further understanding of these genes.

### Case study 3: metabolic genes

ATP citrate lyase (*ACLY*) is a key lipogenic enzyme that catalyzes the synthesis of acetyl-CoA from mitochondria-derived citrate in the cytoplasm, a unique step that links cellular glucose catabolism and lipid synthesis. *ACLY* has been documented as being dramatically upregulated in a number of human carcinoma cell lines with high aerobic glycolytic activities, such as breast and bladder carcinoma [17]. Higher glycolysis is thought to not only provide ATP for the tumor’s high energy demands, but also provide precursors for anabolic processes, including de novo fatty acid synthesis that utilizes acetyl-CoA as the initial precursor. In support of the notion that increased glycolysis contributes to enhanced carcinogenesis by providing self-produced fatty acids for membrane biosynthesis, suppression of *ACLY* through genetic and pharmacological approaches was shown to result in a remarkable inhibition of proliferation and differentiation of certain carcinoma cell lines [18]. However, it is currently unclear whether the dysregulated expression of *ACLY* contributes to the initiation, progression, and metastasis of HCC. Interestingly, *ACLY* is located in chromosome 17q, a frequently amplified region in HCC tissues [19]. By searching Liverome, we found that four gene expression studies have reported an up-regulation of *ACLY* in HCC (Figure S5, Additional File 2). Combined with the known functions of *ACLY* in other solid tumors, the information retrieved from Liverome suggests that *ACLY* can be a potential target for HCC treatment. In addition, through a Liverome search, we found that the glucose transporter *SLC2A1* (also called *GLUT1*), another important player in cancer metabolism [20], was upregulated in AFP-positive HCC tissues and was downregulated in AFP-negative HCC tissues (Figure S6, Additional File 2). This piece of information raises the possibility that different HCC patient segments may have different *GLUT1* expression level.

### Case study 4: co-occurrence network analysis

**Figure 5:**
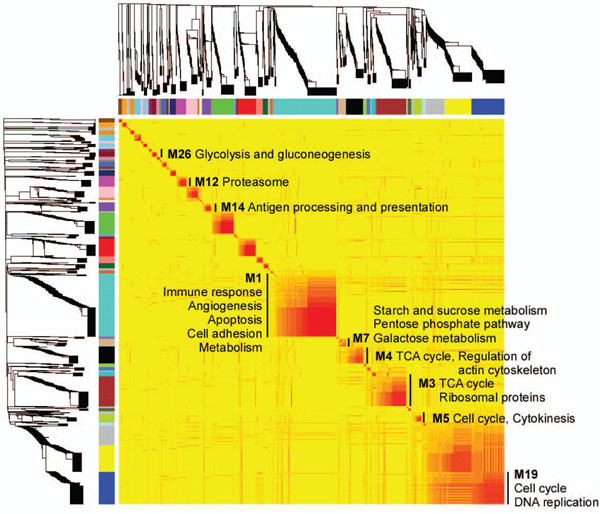
**Co-occurrence network of genes in Liverome.** Shown here is a matrix representation of the co-occurrence network, where genes are represented on rows and columns in the same order. Elements in the matrix are color coded according to the similarity between corresponding pairs of genes. Genes that are grouped into modules are indicated by the color bars and by the associated clustering trees. Blocks along the diagonal indicate that the genes in the same module are more interconnected than those between modules. Details on the plot can be found in the documentation of the WGCNA package. The enriched biological pathways for selected modules are also shown. The modules are numbered as indicated in Additional File 4.

Lastly, we applied a systems biology approach to study the relationship among the genes collected in Liverome and whether such relationship recapitulates known HCC biology. Toward this end, we constructed a gene co-occurrence network consisting of all 6,329 genes in the 92 gene signatures in Liverome 2010 version. Briefly, each gene *i* in Liverome was associated with a binary vector *g_i_* of length *N* (=92; the total number of gene lists). Each element *g_ij_* was set to 1 if the gene *i* belongs to the gene list *j* and 0 otherwise. We then constructed a co-occurrence network in which each node represents a gene and each edge represents a gene pair’s similarity as measured by the Jaccard similarity coefficient between the two gene vectors. In other words, a pair of genes is considered similar if the two genes occur together in the same gene list frequently. Using the WGCNA method [21], we then identified 38 gene modules that represent strongly inter-connected genes in the network. More details on the network construction and module identification can be found in Additional File 3. The modules were then searched for enrichment of known biological pathways in MSigDB [22]. Results show that 24 of the 38 modules are statistically significantly enriched in at least one curated pathway (Additional File 4). The enriched modules reveal many interesting pathways that are relevant to cancer and HCC in particular (Figure 5). For example, the M19 and M5 modules are both enriched in cell cycle genes (*p*<0.001 and *p*<0.0002 by Fisher’s exact test). The M19 module contains core cell cycle checkpoint genes such as *CCNA2*, *E2F2*, and *MCM3*, whereas the M5 module includes genes such as *ANLN*, *PRC1*, and *RACGAP1*, which are mostly involved in cytokinesis. Several modules, such as the M26 module (*p*<0.0022), are enriched in glycolysis pathway genes, which have recently been shown to be critical for HCC tumor growth and therapy failure [23,24]. Furthermore, the M1 module, which is the largest module consisting of 1,018 genes, are enriched for a number of important cancer-related pathways including immune response, angiogenesis, apoptosis, cell adhesion, metabolism, among others. The M1 module seems to represent the core HCC machinery that integrates signals from both internal and external of the tumor cell to adapt to the tumor’s needs to grow and migrate. Most of these enriched pathways were also observed in co-expression network modules identified from a gene expression dataset of a large cohort of HCC primary tumors [25]. Taken together, the co-occurrence network analysis recapitulates known HCC biology and demonstrates that the collective information captured in Liverome is valuable.

## Conclusions

Liverome database provides a comprehensive collection of well-curated HCC gene signatures and straightforward interfaces for gene search and signature comparison as well. The collected signatures were subjected to a thorough manual post-processing before inclusion into the database. Deriving a uniformly formatted and informative form of gene signatures with self-contained context information was the key aim of the post-processing. Characteristics of the gene signatures were also manually annotated to produce an informative signature naming and a detailed yet concise itemized summary. The web interface of Liverome displays gene search result in the most readable way, organizes the diverse gene signatures according to their functional categories for guided browsing, and enables their comparison to identify and prioritize common genes. Using Liverome, liver cancer researchers can have convenient access to a comprehensive and well-curated set of HCC gene signatures accumulated from a decade of molecular profiling studies on HCC. Liverome is most useful to retrieve detailed supporting evidence on a user-specified candidate gene from the published microarray and proteomic studies in HCC. The development of Liverome was initiated to compare our own gene signatures derived from microarray experiments done on a large HCC cohort in Korea (through “The 21C Frontier Functional Human Genome Project of Korea”) with publicly reported gene signatures. We found Liverome database useful for prioritizing the genes present in our own signatures for further in-depth studies. In the future, we will continuously expand Liverome’s signature collection once a year.

## Availability and requirements

The database is available at http://liverome.kobic.re.kr without login requirement. All contents in Liverome are freely available for download and on-site use without restriction.

## Competing interests

The authors declare that they have no competing interests.

## Authors' contributions

JB, HY, and SH conceived of the study. LL, KW, and SH collected gene signatures. LL and SH post-processed and annotated the gene signatures. LL developed the database and web interface. SH modified the database and web interface. KW and SH checked the web site for errors and improvements. KL, YJJ, and YIY guided the database and web interface design. KW, GL, ZX, YW, JX, SS, and CK performed case studies and wrote the relevant sections in the manuscript. GL, HY, and SH wrote the manuscript. DP and JB revised the manuscript critically. All authors read and approved the final manuscript.

## Supplementary Material

Additional file 1 **Supplementary Tables** This document contains all supplementary tables (Tables S1 through S4). Click here for file

Additional file 2 **Supplementary Figures** This document contains all supplementary figures (Figures S1 through S6). Click here for file

Additional file 3 **Supplementary Methods** This document contains methods for co-occurrence network construction and module identification. Click here for file

Additional file 4 **Supplementary Excel File** This Excel file contains the list of modules in the co-expression network and their enriched pathways. Click here for file
