# Supplementary material for: Liverome: a curated database of liver cancer-related gene signatures with self-contained context information
Source: BMC Genomics. 2011 Nov 30;12(Suppl 3):S3. doi: 10.1186/1471-2164-12-S3-S3 (PMC3333186; doi:10.1186/1471-2164-12-S3-S3)
Supplement: Additional file 1 — Supplementary Tables This document contains all supplementary tables (Tables S1 through S4). [file 1471-2164-12-S3-S3-S1.pdf]

Additional file 1: contains all supplementary tables

Liverome: a curated database of liver cancer-related gene signatures with self-contained context information

Content

Table S1. Listing of 143 gene signatures derived from 98 studies (page 2)

Table S2: Number of collected signatures and genes, summarized by functional categories (page 12)

Table S3: Top 22 most frequently occurring genes (page 13)

Table S4. Genes that are frequently mutated in HCC as identified from COSMIC database, and their gene signature hits (page 14)

Table S1. Listing of 143 gene signatures derived from 98 studies.

	Author	Year	Journal	Title and link to PubMed	Type of study	# lists	Characteristics of signatures	Category
1	Archer	2009	Cancer Epidemiol Biomarkers Prev	Identifying genes for establishing a multigenic test for hepatocellular carcinoma surveillance in hepatitis C virus-positive cirrhotic patients	Transcriptomics	1	Cirrhosis with cocommitant HCC vs Cirrhosis without HCC	**Category 3:** Lists related to cirrhosis and dysplasia
2	Boyault	2007	Hepatology	Transcriptome classification of HCC is related to gene alterations and to new therapeutic targets	Transcriptomics	9	G1 and G2 and G3 subgroup-specific genes	**Category 9:** Subgroup-specific lists
							G1 and G2 subgroup-specific genes	
							G1 subgroup-specific genes	
							G2 and G3 subgroup-specific genes	
							G2 subgroup-specific genes	
							G3 subgroup-specific genes	
							G5 and G6 subgroup-specific genes	
							G5 subgroup-specific genes	
							G6 subgroup-specific genes	
3	Braconi	2009	Cancer	Candidate therapeutic agents for hepatocellular cancer can be identified from phenotype-associated gene expression signatures	Transcriptomics	1	Vascular invasive HCC vs Non-invasive HCC	**Category 6:** Lists related to invasion and metastasis
4	Breuhahn	2004	Cancer Res	Molecular profiling of human hepatocellular carcinoma defines mutually exclusive interferon regulation and insulin-like growth factor II overexpression	Transcriptomics	1	Poorly differentiated HCC vs Well differentiated HCC	**Category 5:** Lists related to differentiation
5	Budhu	2006	Cancer Cell	Prediction of venous metastases, recurrence, and prognosis in hepatocellular carcinoma based on a unique immune response signature of the liver microenvironment	Transcriptomics	1	Genes associated with intrahepatic metastasis	**Category 6:** Lists related to invasion and metastasis
6	Calvisi	2007	J Clin Invest	Mechanistic and prognostic significance of aberrant methylation in the molecular pathogenesis of human hepatocellular carcinoma	Others	1	Tumor suppressor genes with hypermethylated promoter in HCC	**Category 10:** Lists of other nature
7	Chaerkady	2008	J Proteome Res	A quantitative proteomic approach for identification of potential biomarkers in	Proteomics	1	Tumor vs Non-tumor	**Category 1:** Lists with tumor-specific expression

				hepatocellular carcinoma				changes
8	Chan	2006	Mod Pathol	Positional expression profiling indicates candidate genes in deletion hotspots of hepatocellular carcinoma	Transcriptomics	1	Tumor vs Non-tumor: down-regulated genes only	**Category 1:** Lists with tumor-specific expression changes
9	Chan	2009	Oncogene	Thyroid hormone receptor mutants implicated in human hepatocellular carcinoma display an altered target gene repertoire	Transcriptomics	1	Genes deregulated by mutations in thyroid hormone receptors	**Category 8:** Lists modulated by a single gene/protein factor
10	Chen	2002	Mol Biol Cell	Gene expression patterns in human liver cancers	Transcriptomics	1	Tumor vs Non-tumor	**Category 1:** Lists with tumor-specific expression changes
11	Chiang	2008	Cancer Res	Focal gains of VEGFA and molecular classification of hepatocellular carcinoma	Transcriptomics	5	Genes specific to Chr7 polysomy subgroup	**Category 9:** Subgroup-specific lists
							Genes specific to CTNNB1-associated subgroup	
							Genes specific to interferon-related subgroup	
							Genes specific to proliferation subgroup	
							Genes specific to unannotated subgroup	
12	Chuma	2003	Hepatology	Expression profiling in multistage hepatocarcinogenesis: identification of HSP70 as a molecular marker of early hepatocellular carcinoma	Transcriptomics	2	Early HCC vs Non-tumor	**Category 5:** Lists related to differentiation
							Progressed HCC vs Early HCC	
13	Chung	2002	Mol Cells	Gene expression profile analysis in human hepatocellular carcinoma by cDNA microarray	Transcriptomics	2	Tumor vs Non-tumor	**Category 1:** Lists with tumor-specific expression changes
							Solid HCC vs Grandular HCC	**Category 5:** Lists related to differentiation
14	Cui	2010	Int J Cancer	EFNA1 ligand and its receptor EphA2: potential biomarkers for hepatocellular carcinoma	Transcriptomics	3	Tumor vs Non-tumor: secretory or membrane-associated genes only	**Category 1:** Lists with tumor-specific expression changes
							Well differentiated HCC vs Cirrhosis: secretory or membrane-associated genes only	**Category 3:** Lists related to cirrhosis and dysplasia
							Poorly differentiated HCC vs Well differentiated HCC: secretory or	**Category 5:** Lists related to differentiation

							membrane-associated genes only	
15	De Giorgi	2009	J Transl Med	Gene profiling, biomarkers and pathways characterizing HCV-related hepatocellular carcinoma	Transcriptomics	3	Non-tumor vs Normal liver	**Category 1:** Lists with tumor-specific expression changes
							Tumor vs Non-tumor	
							Tumor vs Normal liver	
16	Delpuech	2002	Oncogene	Identification, using cDNA macroarray analysis, of distinct gene expression profiles associated with pathological and virological features of hepatocellular carcinoma	Transcriptomics	6	Tumor vs Non-tumor: HBV-positive samples only	**Category 1:** Lists with tumor-specific expression changes
							Tumor vs Non-tumor	
							Genes specifically regulated in cirrhotic HCC	**Category 3:** Lists related to cirrhosis and dysplasia
							Genes specifically regulated in non-cirrhotic HCC	
							Genes specifically regulated in moderately-to-poorly differentiated HCC	**Category 5:** Lists related to differentiation
							Genes specifically regulated in well differentiated HCC	
17	Dong	2009	BMC Med Genomics	Gene expression profile analysis of human hepatocellular carcinoma using SAGE and LongSAGE	Transcriptomics	1	Tumor vs Normal liver	**Category 1:** Lists with tumor-specific expression changes
18	Goldenberg	2002	Mol Carcinog	Analysis of differentially expressed genes in hepatocellular carcinoma using cDNA arrays	Transcriptomics	1	Tumor vs Non-tumor	**Category 1:** Lists with tumor-specific expression changes
19	Han	2000	Biochem Biophys Res Commun	Selective transcriptional regulations in the human liver cell by hepatitis B viral X protein	Transcriptomics	1	Genes regulated by HBx protein	**Category 4:** Lists related to etiology
20	Hoshida	2008	N Engl J Med	Gene expression in fixed tissues and outcome in hepatocellular carcinoma	Transcriptomics	1	Genes associated with survival	**Category 2:** Lists related to survival and recurrence
21	Hoshida	2009	Cancer Res	Integrative transcriptome analysis reveals common molecular subclasses of human hepatocellular carcinoma	Transcriptomics	3	Genes specific to S1 subgroup	**Category 9:** Subgroup-specific lists
							Genes specific to S2 subgroup	
							Genes specific to S3 subgroup	
22	Hsu	2007	BMC Bioinformatics	Detection of the inferred interaction network in hepatocellular carcinoma from EHCO (Encyclopedia of Hepatocellular Carcinoma genes Online)	Others	1	HCC-related genes from PubMed text mining	**Category 10:** Lists of other nature
23	Hu	2004	Oncogene	Association of Vimentin overexpression and hepatocellular carcinoma metastasis	Transcriptomics	1	Metastatic HCC cell line vs Primary HCC cell line	**Category 6:** Lists related to invasion and metastasis

24	Iizuka	2002	Cancer Res	Comparison of gene expression profiles between hepatitis B virus- and hepatitis C virus-infected hepatocellular carcinoma by oligonucleotide microarray data on the basis of a supervised learning method	Transcriptomics	1	HBV-tumor vs HCV-tumor	**Category 4:** Lists related to etiology
25	Iizuka	2003	Lancet	Oligonucleotide microarray for prediction of early intrahepatic recurrence of hepatocellular carcinoma after curative resection	Transcriptomics	1	Early intrahepatic recurrence group vs Non-recurrence group	**Category 2:** Lists related to survival and recurrence
26	Iizuka	2003	Oncogene	Differential gene expression in distinct virologic types of hepatocellular carcinoma: association with liver cirrhosis	Transcriptomics	2	HBV-HCC with cirrhosis vs HBV-HCC without cirrhosis<br>HCV-HCC with cirrhosis vs HCV-HCC without cirrhosis	**Category 3:** Lists related to cirrhosis and dysplasia
27	Iizuka	2005	FEBS Lett	Self-organizing-map-based molecular signature representing the development of hepatocellular carcinoma	Transcriptomics	2	Moderately differentiated HCC vs Well differentiated HCC<br>Poorly differentiated HCC vs Moderately differentiated HCC	**Category 5:** Lists related to differentiation<br>**Category 5:** Lists related to differentiation
28	Iizuka	2006	J Cancer Res Clin Oncol	Involvement of c-myc-regulated genes in hepatocellular carcinoma related to genotype-C hepatitis B virus	Transcriptomics	1	Tumor vs Non-tumor	**Category 1:** Lists with tumor-specific expression changes
29	Iizuka	2006	Oncol Rep	Different molecular pathways determining extrahepatic and intrahepatic recurrences of hepatocellular carcinoma	Transcriptomics	1	Extrahepatic recurrence group vs Non-recurrence group	**Category 2:** Lists related to survival and recurrence
30	Kato	2005	Nucleic Acids Res	Cancer gene expression database (CGED): a database for gene expression profiling with accompanying clinical information of human cancer tissues	Transcriptomics	2	Tumor vs Non-tumor<br>HBV-tumor vs HCV-tumor	**Category 1:** Lists with tumor-specific expression changes<br>**Category 4:** Lists related to etiology
31	Kim	2002	Electrophoresis	Proteome analysis of human liver tumor tissue by two-dimensional gel electrophoresis and matrix assisted laser desorption/ionization-mass spectrometry for identification of disease-related proteins	Proteomics	2	Tumor vs Non-tumor<br>Liver-expressed proteins	**Category 1:** Lists with tumor-specific expression changes<br>**Category 10:** Lists of other nature
32	Kim	2003	Clin Cancer Res	Comparison of proteome between hepatitis B virus- and hepatitis C virus-associated hepatocellular carcinoma	Proteomics	2	Differentially regulated proteins depending on viral infection status<br>Differentially regulated proteins regardless of viral infection status	**Category 4:** Lists related to etiology
33	Kim	2004	Biochim Biophys	Feature genes of hepatitis B virus-positive	Transcriptomics	1	Tumor vs Non-tumor	**Category 1:** Lists with

			Acta	hepatocellular carcinoma, established by its molecular discrimination approach using prediction analysis of microarray				tumor-specific expression changes
34	Kim	2004	Hepatology	Cancer-associated molecular signature in the tissue samples of patients with cirrhosis	Transcriptomics	1	Early diagnostic markers	**Category 3:** Lists related to cirrhosis and dysplasia
35	Kurokawa	2003	J Hepatol	Molecular features of non-B, non-C hepatocellular carcinoma: a PCR-array gene expression profiling study	Transcriptomics	2	Non-tumor vs Normal liver<br>Tumor vs Non-tumor	**Category 1:** Lists with tumor-specific expression changes
36	Kurokawa	2004	Clin Cancer Res	Molecular prediction of response to 5-fluorouracil and interferon-alpha combination chemotherapy in advanced hepatocellular carcinoma	Transcriptomics	1	Genes predicting chemotherapeutic responses	**Category 10:** Lists of other nature
37	Kurokawa	2004	J Hepatol	Molecular-based prediction of early recurrence in hepatocellular carcinoma	Transcriptomics	1	Early recurrence group vs Non-recurrence group	**Category 2:** Lists related to survival and recurrence
38	Kurokawa	2006	Int J Oncol	Central genetic alterations common to all HCV-positive, HBV-positive and non-B, non-C hepatocellular carcinoma: a new approach to identify novel tumor markers	Transcriptomics	1	Differentially expressed genes regardless of viral infection status	**Category 4:** Lists related to etiology
39	Lau	2006	Oncogene	Clusterin plays an important role in hepatocellular carcinoma metastasis	Transcriptomics	1	Genes regulated by clusterin	**Category 8:** Lists modulated by a single gene/protein factor
40	Lee	2003	Biotechnol Prog	Discovery of differentially expressed genes related to histological subtype of hepatocellular carcinoma	Transcriptomics	1	Discriminatory genes between poorly and moderately differentiated tumors	**Category 5:** Lists related to differentiation
41	Lee	2004	Hepatology	Classification and prediction of survival in hepatocellular carcinoma by gene expression profiling	Transcriptomics	1	Genes associated with survival	**Category 2:** Lists related to survival and recurrence
42	Lee	2004	Nat Genet	Application of comparative functional genomics to identify best-fit mouse models to study human cancer	Transcriptomics	1	Poorer survival group vs Better survival group	**Category 2:** Lists related to survival and recurrence
43	Lee	2008	Cancer Res	Functional and clinical evidence for NDRG2 as a candidate suppressor of liver cancer metastasis	Transcriptomics	1	Tumor vs Non-tumor: down-regulated genes only	**Category 1:** Lists with tumor-specific expression changes
44	Lee	2008	Clin Cancer Res	Identification of cystatin B as a potential serum marker in hepatocellular carcinoma	Transcriptomics	1	Tumor vs Non-tumor	**Category 1:** Lists with tumor-specific expression changes
45	Lee	2008	World J Gastroenterol	Distinct expression patterns in hepatitis B virus- and hepatitis C virus-infected hepatocellular carcinoma.	Transcriptomics	1	HBV-tumor vs HCV-tumor	**Category 4:** Lists related to etiology

46	Li	2002	J Cancer Res Clin Oncol	Discovery and analysis of hepatocellular carcinoma genes using cDNA microarrays	Transcriptomics	1	Tumor vs Normal liver	**Category 1:** Lists with tumor-specific expression changes
47	Li	2003	J Cancer Res Clin Oncol	Establishment of a hepatocellular carcinoma cell line with unique metastatic characteristics through in vivo selection and screening for metastasis-related genes through cDNA microarray	Transcriptomics	1	High metastatic potential cell line vs Low metastatic potential cell line	**Category 6:** Lists related to invasion and metastasis
48	Li	2004	Mol Cell Proteomics	Accurate qualitative and quantitative proteomic analysis of clinical hepatocellular carcinoma using laser capture microdissection coupled with isotope-coded affinity tag and two-dimensional liquid chromatography mass spectrometry	Proteomics	1	Tumor vs Non-tumor	**Category 1:** Lists with tumor-specific expression changes
49	Li	2005	Proteomics	Proteomic analysis of hepatitis B virus-associated hepatocellular carcinoma: Identification of potential tumor markers	Proteomics	1	Tumor vs Non-tumor	**Category 1:** Lists with tumor-specific expression changes
50	Liang	2005	Proteomics	Proteome analysis of human hepatocellular carcinoma tissues by two-dimensional difference gel electrophoresis and mass spectrometry	Proteomics	2	Poorly differentiated tumor vs Non-tumor<br>Well differentiated tumor vs Non-tumor	**Category 5:** Lists related to differentiation
51	Liao	2008	Oncogene	Identification of SOX4 target genes using phylogenetic footprinting-based prediction from expression microarrays suggests that overexpression of SOX4 potentiates metastasis in hepatocellular carcinoma	Transcriptomics	1	Genes over-expressed in metastatic HCC and containing SOX4-binding sites	**Category 6:** Lists related to invasion and metastasis
52	Lim	2002	BBRC	Proteome analysis of hepatocellular carcinoma	Proteomics	1	Tumor vs Non-tumor vs Cirrhotic tissue	**Category 3:** Lists related to cirrhosis and dysplasia
53	Mas	2004	Liver Transpl	Hepatocellular carcinoma in HCV-infected patients awaiting liver transplantation: genes involved in tumor progression	Transcriptomics	1	Late cirrhosis vs Early cirrhosis	**Category 3:** Lists related to cirrhosis and dysplasia
54	Mas	2009	Mol Med	Genes involved in viral carcinogenesis and tumor initiation in hepatitis C virus-induced hepatocellular carcinoma	Transcriptomics	1	HCV-cirrhosis with concommitant HCC vs HCV-cirrhosis without HCC	**Category 3:** Lists related to cirrhosis and dysplasia
55	Matoba	2005	Int J Cancer	Tumor HLA-DR expression linked to early intrahepatic recurrence of hepatocellular carcinoma	Transcriptomics	1	Early intrahepatic recurrence group vs Non-recurrence group	**Category 2:** Lists related to survival and recurrence
56	Midorikawa	2002	Jpn J Cancer Res	Identification of genes associated with dedifferentiation of hepatocellular carcinoma with expression profiling	Transcriptomics	1	Well differentiated HCC vs Moderately differentiated HCC	**Category 5:** Lists related to differentiation

				analysis				
57	Muller	2005	Cell Death Differ	TAp73/Delta Np73 influences apoptotic response, chemosensitivity and prognosis in hepatocellular carcinoma	Transcriptomics	1	Genes up-regulated by TAp73-beta	**Category 8:** Lists modulated by a single gene/protein factor
58	Nam	2005	Hepatology	Molecular changes from dysplastic nodule to hepatocellular carcinoma through gene expression profiling	Transcriptomics	2	Early-stage genes	**Category 5:** Lists related to differentiation
							Late-stage genes	
59	Neo	2004	Hepatology	Identification of discriminators of hepatoma by gene expression profiling using a minimal dataset approach	Transcriptomics	1	Tumor vs Non-tumor	**Category 1:** Lists with tumor-specific expression changes
60	Nquyen	2006	Virology	Hepatitis C virus core protein induces expression of genes regulating immune evasion and anti-apoptosis in hepatocytes	Transcriptomics	1	Genes regulated by HCV core protein	**Category 4:** Lists related to etiology
61	Okabe	2001	Cancer Res	Genome-wide analysis of gene expression in human hepatocellular carcinomas using cDNA microarray: identification of genes involved in viral carcinogenesis and tumor progression	Transcriptomics	4	Tumor vs Non-tumor	**Category 1:** Lists with tumor-specific expression changes
							HBV-tumor vs HCV-tumor	**Category 4:** Lists related to etiology
							HCC progression related genes	**Category 5:** Lists related to differentiation
							Vascular invasive HCC vs Non-invasive HCC	**Category 6:** Lists related to invasion and metastasis
62	Okada	2003	FEBS Lett	Gene expression profile linked to p53 status in hepatitis C virus-related hepatocellular carcinoma.	Transcriptomics	1	Genes deregulated by mutations in p53	**Category 8:** Lists modulated by a single gene/protein factor
63	Okamoto	2006	Ann Surg Oncol	Specific gene-expression profiles of noncancerous liver tissue predict the risk for multicentric occurrence of hepatocellular carcinoma in hepatitis C virus-positive patients	Transcriptomics	1	Predictive marker genes for multicentric hepatocarcinogenesis	**Category 2:** Lists related to survival and recurrence
64	Otsuka	2003	BBRC	Differential cellular gene expression induced by hepatitis B and C viruses	Transcriptomics	1	Genes regulated by HBV-transfection	**Category 4:** Lists related to etiology
65	Patil	2005	Oncogene	An integrated data analysis approach to characterize genes highly expressed in hepatocellular carcinoma	Transcriptomics	1	Tumor vs Non-tumor	**Category 1:** Lists with tumor-specific expression changes
66	Saito	2008	Cancer Sci	Molecular background of alpha-fetoprotein in liver cancer cells as revealed by global RNA expression analysis	Transcriptomics	1	Genes co-expressed with AFP	**Category 10:** Lists of other nature

67	Shi	2005	Br J Cancer	Identification and analysis of tumour-associated antigens in hepatocellular carcinoma	Transcriptomics	1	Genes encoding HCC-related antigens	**Category 10:** Lists of other nature
68	Shirota	2001	Hepatology	Identification of differentially expressed genes in hepatocellular carcinoma with cDNA microarrays	Transcriptomics	1	Tumor vs Non-tumor	**Category 1:** Lists with tumor-specific expression changes
69	Simon	2010	Langenbecks Arch Surg	Deregulation of HIF1-alpha and hypoxia-regulated pathways in hepatocellular carcinoma and corresponding non-malignant liver tissue--influence of a modulated host stroma on the prognosis of HCC	Transcriptomics	1	Genes deregulated by HIF1A modulation	**Category 8:** Lists modulated by a single gene/protein factor
70	Skawran	2008	Mod Pathol	Loss of 13q is associated with genes involved in cell cycle and proliferation in dedifferentiated hepatocellular carcinoma	Transcriptomics	2	Poorly differentiated HCC vs Well/Moderately differentiated HCC<br>HCC with 13q loss vs HCC without 13q loss	**Category 5:** Lists related to differentiation<br>**Category 7:** Lists related to genomic alterations
71	Skawran	2008	Mod Pathol	Gene expression profiling in hepatocellular carcinoma: upregulation of genes in amplified chromosome regions	Transcriptomics	1	Genes up-regulated in HCC compared to HCA, and located in amplified chromosomal regions	**Category 7:** Lists related to genomic alterations
72	Sun	2005	Mol Cell Proteomics	Proteome analysis of hepatocellular carcinoma by two-dimensional difference gel electrophoresis: novel protein markers in hepatocellular carcinoma tissues	Proteomics	1	Tumor vs Non-tumor	**Category 1:** Lists with tumor-specific expression changes
73	Sun	2008	J Proteome Res	Quantitative proteomic signature of liver cancer cells: tissue transglutaminase 2 could be a novel protein candidate of human hepatocellular carcinoma	Proteomics	2	AFP-deficient tumor vs Normal liver<br>AFP-producing tumor vs Normal liver	**Category 10:** Lists of other nature
74	Tackels-Horne	2001	Cancer	Identification of differentially expressed genes in hepatocellular carcinoma and metastatic liver tumors by oligonucleotide expression profiling	Transcriptomics	2	Tumor vs Normal liver<br>Metastatic tumor vs Normal liver	**Category 1:** Lists with tumor-specific expression changes<br>**Category 6:** Lists related to invasion and metastasis
75	Tanaka	2008	Br J Surg	Aurora kinase B is a predictive factor for the aggressive recurrence of hepatocellular carcinoma after curative hepatectomy.	Transcriptomics	1	Aggressive recurrence group vs Non-aggressive recurrence or Non-recurrence groups	**Category 2:** Lists related to survival and recurrence
76	Tanaka	2010	Surgery	Gene-expression phenotypes for vascular invasiveness of hepatocellular carcinomas	Transcriptomics	2	Genes associated with development of vascular invasion<br>Macroscopic invasion group vs Non-invasion group	**Category 6:** Lists related to invasion and metastasis

77	Tannapfel	2003	J Pathol	Identification of novel proteins associated with hepatocellular carcinomas using protein microarrays	Proteomics	1	Tumor vs Normal liver	**Category 1:** Lists with tumor-specific expression changes
78	Tominaru	2010	Br J Cancer	Insulin-like growth factor-binding protein 7 alters the sensitivity to interferon-based anticancer therapy in hepatocellular carcinoma cells	Transcriptomics	1	INFalpha-tolerant HCC cell line vs Parental HCC cell line	**Category 10:** Lists of other nature
79	Tsai	2006	J Biomed Sci	Gene expression analysis of human hepatocellular carcinoma by using full-length cDNA library	Transcriptomics	1	Genes under-expressed in tumor and located within frequently deleted loci	**Category 7:** Lists related to genomic alterations
80	Tsuchiya	2010	Mol Cancer	Gene expression in nontumoral liver tissue and recurrence-free survival in hepatitis C virus-positive hepatocellular carcinoma	Transcriptomics	1	Predictive marker genes for recurrence	**Category 2:** Lists related to survival and recurrence
81	Tsunedomi	2006	Int J Oncol	Identification of ID2 associated with invasion of hepatitis C virus-related hepatocellular carcinoma by gene expression profile	Transcriptomics	1	HCC with portal vein invasion vs HCC without portal vein invasion	**Category 6:** Lists related to invasion and metastasis
82	Wang	2004	Br J Cancer	Genomic analysis reveals RhoC as a potential marker in hepatocellular carcinoma with poor prognosis	Transcriptomics	1	Solitary large HCC vs Nodular HCC	**Category 10:** Lists of other nature
83	Wang	2007	Clin Cancer Res	Identification and validation of a novel gene signature associated with the recurrence of human hepatocellular carcinoma	Transcriptomics	1	Recurrence group vs Non-recurrence group	**Category 2:** Lists related to survival and recurrence
84	Wong	2005	Clin Cancer Res	Transcriptional profiling identifies gene expression changes associated with IFN-alpha tolerance in hepatitis C-related hepatocellular carcinoma cells	Transcriptomics	1	INFalpha-tolerant HCC vs INFalpha-sensitive HCC	**Category 10:** Lists of other nature
85	Woo	2008	Clin Cancer Res	Gene expression-based recurrence prediction of hepatitis B virus-related human hepatocellular carcinoma	Transcriptomics	1	High recurrence risk group vs Low recurrence risk group	**Category 2:** Lists related to survival and recurrence
86	Woo	2009	Cancer Res	Identification of potential driver genes in human liver carcinoma by genomewide screening	Others	1	Potential driver genes of HCC	**Category 10:** Lists of other nature
87	Wurmbach	2007	Hepatology	Genome-wide molecular profiles of HCV-induced dysplasia and hepatocellular carcinoma	Transcriptomics	5	Cirrhosis vs Normal liver	**Category 3:** Lists related to cirrhosis and dysplasia
							Dysplasia vs Cirrhosis	
							Early HCC vs Dysplasia	
							HCC vs Dysplasia	
							Advanced HCC vs Early HCC	**Category 5:** Lists related to

								differentiation
88	Xu	2001	Cancer Res	Expression profiling suggested a regulatory role of liver-enriched transcription factors in human hepatocellular carcinoma	Transcriptomics	1	Tumor vs Non-tumor	**Category 1:** Lists with tumor-specific expression changes
89	Xu	2001	PNAS	Insight into hepatocellular carcinogenesis at transcriptome level by comparing gene expression profiles of hepatocellular carcinoma with those of corresponding noncancerous liver	Transcriptomics	1	Tumor vs Non-tumor	**Category 1:** Lists with tumor-specific expression changes
90	Yamashita	2001	BBRC	Serial analysis of gene expression in chronic hepatitis C and hepatocellular carcinoma	Transcriptomics	2	Non-tumor vs Normal liver<br>Tumor vs Normal liver	**Category 1:** Lists with tumor-specific expression changes
91	Yang	2005	BBRC	Integrative genomics based identification of potential human hepatocarcinogenesis-associated cell cycle regulators: RHAMM as an example	Transcriptomics	1	Cell cycle-regulated, over-expressed, and vertebrate-specific genes	**Category 10:** Lists of other nature
92	Yasen	2009	Cancer Sci	Expression of Aurora B and alternative variant forms in hepatocellular carcinoma and adjacent tissue	Transcriptomics	1	Genes deregulated by expression of AURKB splicing variant 2	**Category 8:** Lists modulated by a single gene/protein factor
93	Ye	2003	Nat Med	Predicting hepatitis B virus-positive metastatic hepatocellular carcinomas using gene expression profiling and supervised machine learning	Transcriptomics	1	Genes associated with intrahepatic metastasis	**Category 6:** Lists related to invasion and metastasis
94	Yeh	2003	Oncogene	Antisense overexpression of BMAL2 enhances cell proliferation	Transcriptomics	1	Tumor vs Non-tumor	**Category 1:** Lists with tumor-specific expression changes
95	Yokoo	2007	Cancer Sci	Protein expression associated with early intrahepatic recurrence of hepatocellular carcinoma after curative surgery.	Proteomics	1	Early intrahepatic recurrence group vs Non-recurrence group	**Category 2:** Lists related to survival and recurrence
96	Yokoyama	2004	Proteomics	Proteomic profiling of proteins decreased in hepatocellular carcinoma from patients infected with hepatitis C virus	Proteomics	1	Tumor vs Non-tumor	**Category 1:** Lists with tumor-specific expression changes
97	Yoshioka	2009	Eur J Cancer	Molecular prediction of early recurrence after resection of hepatocellular carcinoma	Transcriptomics	1	Predictive marker genes for early intrahepatic recurrence	**Category 2:** Lists related to survival and recurrence
98	Zekri	2008	BMC Res Notes	Genetic profile of Egyptian hepatocellular-carcinoma associated with hepatitis C virus Genotype 4 by 15 K cDNA microarray: preliminary study	Transcriptomics	1	Tumor vs Non-tumor	**Category 1:** Lists with tumor-specific expression changes

Table S2. Number of collected signatures and genes, summarized by functional categories.

Category	Number of signatures	Number of unique studies	Number of unique genes
Category 1: Lists with tumor-specific expression changes	37	32	3660
Category 2: Lists related to survival and recurrence	14	14	934
Category 3: Lists related to cirrhosis and dysplasia	14	9	559
Category 4: Lists related to etiology	10	9	453
Category 5: Lists related to differentiation	18	13	628
Category 6: Lists related to invasion and metastasis	11	10	902
Category 7: Lists related to genomic alterations	4	4	104
Category 8: Lists modulated by a single gene/protein factor	6	6	256
Category 9: Subgroup-specific lists	17	3	2046
Category 10: Lists of other nature	12	11	1776
**Total**	**143 lists**	**98 studies**	**6927 genes**

Table S3. Top 22 most frequently occurring genes. This table was generated by selecting all 143 Liverome-collected gene signatures on the "View/Compare Lists" menu. Default ordering was applied; descending sort on occurrence frequency, followed by ascending sort on gene symbol.

Occurrence (out of 143 lists)	Gene symbol	Entrez Gene ID	Gene name
23	ECHS1	1892	enoyl CoA hydratase, short chain, 1, mitochondrial
18	ADH1B	125	alcohol dehydrogenase 1B (class I), beta polypeptide
17	GPC3	2719	glypican 3
16	ALB	213	albumin
	BHMT	635	betaine--homocysteine S-methyltransferase
	PLG	5340	plasminogen
	VIM	7431	vimentin
15	RGN	9104	regucalcin (senescence marker protein-30)
	TF	7018	transferrin
14	FABP1	2168	fatty acid binding protein 1, liver
	HPD	3242	4-hydroxyphenylpyruvate dioxygenase
13	ACADSB	36	acyl-CoA dehydrogenase, short/branched chain
	CAT	847	catalase
	MTHFD1	4522	methylenetetrahydrofolate dehydrogenase (NADP+ dependent) 1
	RPSA	3921	ribosomal protein SA
	SLC22A1	6580	solute carrier family 22 (organic cation transporter), member 1
	TDO2	6999	tryptophan 2,3-dioxygenase
12	ADH4	127	alcohol dehydrogenase 4 (class II), pi polypeptide
	CP	1356	ceruloplasmin (ferroxidase)
	CYP2E1	1571	cytochrome P450, family 2, subfamily E, polypeptide 1
	PCK1	5105	phosphoenolpyruvate carboxykinase 1 (soluble)
	SPARC	6678	secreted protein, acidic, cysteine-rich (osteonectin)

Table S4. Genes that are frequently mutated in HCC as identified from COSMIC database, and their gene signature hits.

Symbol	Information from COSMIC			Information from Liverome			
	Mutation freq (%)	Mutated samples	Samples screened	Gene signature hits (from transcriptomic or proteomic studies only)			
HNF1A	27.07	86	318	Saito	(2008)	*Cancer Sci*	Genes co-expressed with AFP
CSF1R	21.43	3	14	Chen	(2002)	*Mol Biol Cell*	Tumor vs Non-tumor
CTNNB1	21.07	604	2866	Delpuech<br>Han<br>Hoshida	(2002)<br>(2000)<br>(2009)	*Oncogene*<br>*BBRC*<br>*Cancer Res*	Genes specifically regulated in non-cirrhotic HCC<br>Genes regulated by HBx protein<br>Genes specific to S2 subgroup
CDKN2A	14.81	72	486	Neo	(2004)	*Hepatology*	Tumor vs Non-tumor
APC	11.54	9	78	NONE			
RB1	9.52	2	21	Kurokawa	(2004)	*J Hepatol*	Early recurrence group vs Non-recurrence group
SMO	7.69	1	13	NONE			
KRAS	7.30	17	233	Boyault<br>Kim<br>Nquyen	(2007)<br>(2004)<br>(2006)	*Hepatology*<br>*Hepatology*<br>*Virology*	G3 subgroup-specific genes<br>Early diagnostic markers<br>Genes regulated by HCV core protein
PTEN	5.94	12	202	Kurokawa<br>Kato	(2003)<br>(2005)	*J Hepatol*<br>*Nucleic Acids Res*	Non-tumor vs Normal liver<br>Tumor vs Non-tumor
PIK3CA	5.88	20	340	NONE			
MET	5.56	3	54	Simon	(2010)	*Langenbecks Arch Surg*	Genes deregulated by HIF1A modulation
