# Supplementary material for: Liverome: a curated database of liver cancer-related gene signatures with self-contained context information
Source: BMC Genomics. 2011 Nov 30;12(Suppl 3):S3. doi: 10.1186/1471-2164-12-S3-S3 (PMC3333186; doi:10.1186/1471-2164-12-S3-S3)
Supplement: Additional file 2 — Supplementary Figures This document contains all supplementary figures (Figures S1 through S6). [file 1471-2164-12-S3-S3-S2.pdf]

Additional file 2: contains all supplementary figures

Liverome: a curated database of liver cancer-related gene signatures with self-contained context information

Content

Figure S1. Occurrence frequency of genes in Liverome-collected signatures (page 2)

Figure S2. The gene search result for GPC3 (page 3)

Figure S3. The gene search result for BHMT (page 4)

Figure S4. The gene search result for MTHFD1 (page 5)

Figure S5. The gene search result for ACLY (page 6)

Figure S6. The gene search result for SLC2A1 (page 7)

Figure S1. Occurrence frequency of genes in Liverome-collected signatures. There are a total of 6,927 genes that are observed in at least one of the 143 signatures collected in Liverome. Among them, 53% of the genes occur in only one of the lists while a few genes occur frequently.

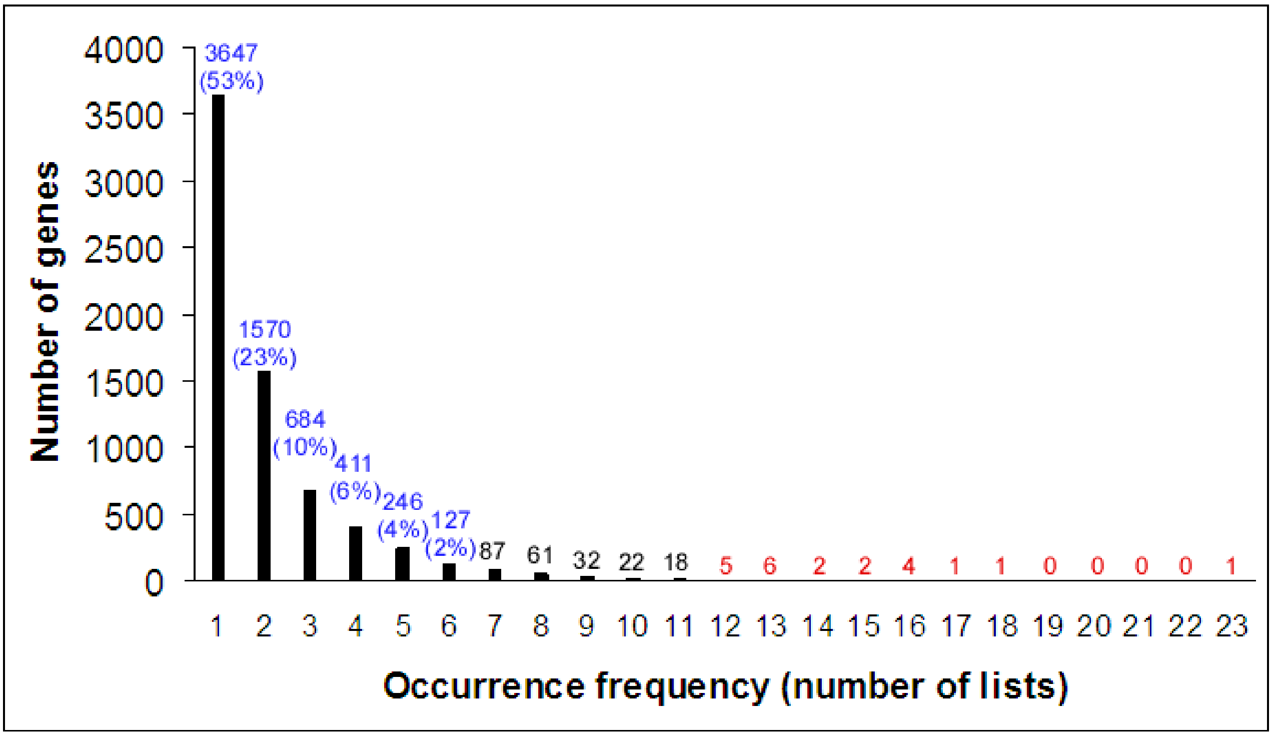

Figure S2. **The gene search result for GPC3.** GPC3 was found to occur in 17 Liverome-collected signatures. Among them, it can be seen that up-regulation of GPC3 is consistently supported by eight signatures derived from comparison of tumor with non-tumor or normal liver, along with large fold change values (marked in blue boxes).

Description of gene list	Evidence	
Neo (2004)<br>*Hepatology*<br>Tumor vs Non-tumor	**Change direction (Tumor/Non-tumor)**	Up
Nam (2005)<br>*Hepatology*<br>Late-stage genes	**Relevant Grade**	Grade 1
	**P-value (t-test)**	3.400E-5
Yamashita (2001)<br>*BBRC*<br>Tumor vs Normal liver	**Fold change (Tumor/Normal)**	15.000 Up
Wurmbach (2007)<br>*Hepatology*<br>HCC vs Dysplasia	**Differentially expressed between**	HCC and dysplasia
Kato (2005)<br>*Nucleic Acids Res*<br>HBV-tumor vs HCV-tumor	**P-value**	0.037
Kato (2005)<br>*Nucleic Acids Res*<br>Tumor vs Non-tumor	**P-value**	2.760E-5
Hoshida (2009)<br>*Cancer Res*<br>Genes specific to S2 subgroup	**Specific to**	S2 subgroup
De Giorgi (2009)<br>*J Transl Med*<br>Tumor vs Normal liver	**Change direction (Tumor/Normal)**	Up
Hsu (2007)<br>*BMC Bioinformatics*<br>HCC-related genes from PubMed text mining	**Related to**	HCC
Tackels-Horne (2001)<br>*Cancer*<br>Tumor vs Normal liver	**Fold change (Tumor/Normal)**	10.700 Up
	**P-value**	0.023
Otsuka (2003)<br>*BBRC*<br>Genes regulated by HBV-transfection	**Fold change (HBV-transfected HepG2.2.15 cell/Parental HepG2 cell)**	3.600 Down
Patil (2005)<br>*Oncogene*<br>Tumor vs Non-tumor	**Fold change (Tumor/Non-tumor)**	45.188 Up
Chen (2002)<br>*Mol Biol Cell*<br>Tumor vs Non-tumor	**Fold change (Tumor/Non-tumor)**	18.296 Up
Kim (2004)<br>*Biochim Biophys Acta*<br>Tumor vs Non-tumor	**Change direction (Tumor/Non-tumor)**	Up
Dong (2009)<br>*BMC Med Genomics*<br>Tumor vs Normal liver	**Fold change (Tumor vs Normal)**	69.000 Up
Saito (2008)<br>*Cancer Sci*<br>Genes co-expressed with AFP	**Correlation coefficient with AFP**	0.931
Chan (2009)<br>*Oncogene*<br>Genes deregulated by mutations in thyroid hormone receptors	**Change direction (Cell line with mutant TR/Cell line with wildtype TR)**	Up

Figure S3. **The gene search result for BHMT.** BHMT was found to occur in 16 Liverome-collected signatures. Among them, it can be seen that down-regulation of BHMT is prevalent in proliferative and poorly differentiated HCCs (marked in blue boxes).

Description of gene list	Evidence	
Iizuka (2006)<br>*J Cancer Res Clin Oncol*<br>Tumor vs Non-tumor	Fold change (Tumor/Non-tumor)	4.476 Down
Kato (2005)<br>*Nucleic Acids Res*<br>Tumor vs Non-tumor	P-value	0.004
Delpuech (2002)<br>*Oncogene*<br>Tumor vs Non-tumor: HBV-positive samples only	Change direction (Tumor/Non-tumor)	Down
Delpuech (2002)<br>*Oncogene*<br>Tumor vs Non-tumor	Change direction (Tumor/Non-tumor)	Down
Breuhahn (2004)<br>*Cancer Res*<br>Poorly differentiated HCC vs Well differentiated HCC	Fold change (PD tumor/WD tumor)	59.700 Down
	Fold change (PD tumor/Normal)	3.700 Down
	Fold change (WD tumor/Normal)	16.000 Up
Sun (2005)<br>*Mol Cell Proteomics*<br>Tumor vs Non-tumor	Fold change (Tumor/Non-tumor)	3.540 Down
	P-value	0.003
Liang (2005)<br>*Proteomics*<br>Poorly differentiated tumor vs Non-tumor	Fold change (PD tumor/Non-tumor)	13.860 Down
	P-value	1.417E-4
Liang (2005)<br>*Proteomics*<br>Well differentiated tumor vs Non-tumor	Fold change (WD tumor/Non-tumor)	5.843 Down
	P-value	0.004
Kim (2003)<br>*Clin Cancer Res*<br>Differentially regulated proteins depending on viral infection status	Cases with >1.5-fold (out of 7 non-B, non-C patients)	6
	Fold change (in non-B, non-C patients) (Tumor/Non-tumor)	1.800 Up
	Cases with >1.5-fold (out of 7 HBV patients)	2
	Fold change (in HBV patients) (Tumor/Non-tumor)	1.700 Up
	Cases with >1.5-fold (out of 7 HCV patients)	None
	Fold change (in HCV patients) (Tumor/Non-tumor)	Not significant
Chaerkady (2008)<br>*J Proteome Res*<br>Tumor vs Non-tumor	Fold change (Tumor/Non-tumor)	2.500 Down
Xu (2001)<br>*PNAS*<br>Tumor vs Non-tumor	Change direction (Tumor/Non-tumor)	Down
Hoshida (2009)<br>*Cancer Res*<br>Genes specific to S3 subgroup	Specific to	S3 subgroup
Chiang (2008)<br>*Cancer Res*<br>Genes specific to unannotated subgroup	SAM score	3.700
	Fold change (Unannotated subgroup/Other subgroups)	2.180 Up
	q-value	0.100
Chiang (2008)<br>*Cancer Res*<br>Genes specific to proliferation subgroup	SAM score	-7.680
	Fold change (Proliferation subgroup/Other subgroups)	6.670 Down
	q-value	0
Hsu (2007)<br>*BMC Bioinformatics*<br>HCC-related genes from PubMed text mining	Related to	HCC
Lee (2008)<br>*Clin Cancer Res*<br>Tumor vs Non-tumor	Fold change (Tumor/Non-tumor)	4.283 Down
	P-value	1.000E-4

Figure S4. **The gene search result for MTHFD1.** MTHFD1 was found to occur in 13 Liverome-collected signatures. Among them, it can be seen that down-regulation of MTHFD1 is prevalent in proliferative and poorly differentiated HCCs (marked in blue boxes).

Description of gene list	Evidence	
Liang (2005) *Proteomics* Poorly differentiated tumor vs Non-tumor	Fold change (PD tumor/Non-tumor)	7.860 Down
	P-value	3.900E-4
Chaerkady (2008) *J Proteome Res* Tumor vs Non-tumor	Fold change (Tumor/Non-tumor)	3.333 Down
Hoshida (2009) *Cancer Res* Genes specific to S3 subgroup	Specific to	S3 subgroup
Chiang (2008) *Cancer Res* Genes specific to proliferation subgroup	SAM score	-8.370
	Fold change (Proliferation subgroup/Other subgroups)	3.450 Down
	q-value	0
Chiang (2008) *Cancer Res* Genes specific to CTNNB1-associated subgroup	SAM score	5.750
	Fold change (CTNNB1 subgroup/Other subgroups)	2.120 Up
	q-value	0.00
Boyault (2007) *Hepatology* G1 and G2 and G3 subgroup-specific genes	Fold change (G1 Tumor/Non-tumor)	5.260 Down
	Fold change (G2 Tumor/Non-tumor)	2.380 Down
	Fold change (G3 Tumor/Non-tumor)	2.380 Down
Kim (2004) *Hepatology* Early diagnostic markers	P-value	5.800E-5
	Fold change (Cirrhosis or HCC/Normal) in high-risk group	1.340 Down
	Fold change (Cirrhosis/Normal) in low-risk group	1.211 Up
Delpuech (2002) *Oncogene* Genes specifically regulated in moderately-to-poorly differentiated HCC	Change direction (Tumor/Non-tumor)	Down
Delpuech (2002) *Oncogene* Tumor vs Non-tumor: HBV-positive samples only	Change direction (Tumor/Non-tumor)	Down
Delpuech (2002) *Oncogene* Tumor vs Non-tumor	Change direction (Tumor/Non-tumor)	Down
Sun (2008) *J Proteome Res* AFP-deficient HCC cell line vs Normal liver cell line	Fold change (AFP- tumor/Normal)	2.140 Down
Chen (2002) *Mol Biol Cell* Tumor vs Non-tumor	Fold change (Tumor/Non-tumor)	2.067 Down
Li (2004) *Mol Cell Proteomics* Tumor vs Non-tumor	Fold change (Tumor/Non-tumor)	3.125 Down

Figure S5. **The gene search result for ACLY.** ACLY was found to occur in 6 Liverome-collected signatures. Among them, it can be seen that up-regulation of ACLY is supported by four signatures that compares tumor with non-tumor (marked in blue boxes).

Description of gene list	Evidence	
Sun (2008)<br>*J Proteome Res*<br>AFP-deficient HCC cell line vs Normal liver cell line	**Fold change (AFP- tumor/Normal)**	2.098 Down
Sun (2008)<br>*J Proteome Res*<br>AFP-producing HCC cell line vs Normal liver cell line	**Fold change (AFP+ tumor/Normal)**	2.307 Down
Patil (2005)<br>*Oncogene*<br>Tumor vs Non-tumor	**Fold change (Tumor/Non-tumor)**	2.409 Up
Chen (2002)<br>*Mol Biol Cell*<br>Tumor vs Non-tumor	**Fold change (Tumor/Non-tumor)**	2.092 Up
Neo (2004)<br>*Hepatology*<br>Tumor vs Non-tumor	**Change direction (Tumor/Non-tumor)**	Up
Boyault (2007)<br>*Hepatology*<br>G1 and G2 and G3 subgroup-specific genes	**Fold change (G1 Tumor/Non-tumor)**<br>**Fold change (G2 Tumor/Non-tumor)**<br>**Fold change (G3 Tumor/Non-tumor)**	2.020 Up<br>2.070 Up<br>2.750 Up

Figure S6. **The gene search result for SLC2A1.** SLC2A1 was found to occur in 5 Liverome-collected signatures. Among them, it can be seen that SLC2A1 is upregulated in AFP-positive HCC tissues and downregulated in AFP-negative HCC tissues (marked in blue boxes).

Description of gene list	Evidence	
Hoshida (2009)<br>*Cancer Res*<br>Genes specific to S1 subgroup	**Specific to**	S1 subgroup
Sun (2008)<br>*J Proteome Res*<br>AFP-deficient HCC cell line vs Normal liver cell line	**Fold change (AFP- tumor/Normal)**	2.966 Down
Sun (2008)<br>*J Proteome Res*<br>AFP-producing HCC cell line vs Normal liver cell line	**Fold change (AFP+ tumor/Normal)**	7.514 Up
Braconi (2009)<br>*Cancer*<br>Vascular invasive HCC vs Non-invasive HCC	**Change direction (Invasive/Non-invasive)**	Up
Liao (2008)<br>*Oncogene*<br>Genes over-expressed in metastatic HCC and containing SOX4-binding sites	**Fold change (Metastatic HCC/Primary HCC)**	2.840 Up
