# Supplementary material for: Liverome: a curated database of liver cancer-related gene signatures with self-contained context information
Source: BMC Genomics. 2011 Nov 30;12(Suppl 3):S3. doi: 10.1186/1471-2164-12-S3-S3 (PMC3333186; doi:10.1186/1471-2164-12-S3-S3)
Supplement: Additional file 3 — Supplementary Methods This document contains methods for co-occurrence network construction and module identification. [file 1471-2164-12-S3-S3-S3.pdf]

Additional file 3

Liverome: a curated database of liver cancer-related gene signatures with self-contained context information

Methods on co-occurrence network construction and module identification

The dataset used for network construction includes 92 gene sets from Liverome. A total of N = 6329 genes are selected that belong to at least one of the 92 gene sets. For any two genes, g_i and g_j, a co-occurrence score is computed as the Jaccard similarity coefficient between the two genes. Specifically, denote

M_{11}: Number of gene sets that g_i and g_j both belong to
M_{01}: Number of gene sets that g_i belongs to but not g_j
M_{10}: Number of gene sets that g_j belongs to but not g_i
M_{00}: Number of gene sets that neither g_i nor g_j belongs to

For every gene pair (g_i, g_j), all gene sets must fall into one of above four categories, meaning that $M_{11} + M_{01} + M_{10} + M_{00} = N$, where N is the total number of gene sets. The Jaccard similarity coefficient, J, is defined as:

$$J = \frac{M_{11}}{M_{10} + M_{01} + M_{11}}$$

The co-occurrence network can be then constructed as an adjacency matrix, A, where $A(i,j) = J(g_i, g_j)$, $i=1,2,\ldots,N$ and $j=1,2,\ldots N$. Conceptually, each gene is represented by a node in the network and edges between the nodes are weighted by their co-occurrence similarity. In such a network, a group of strongly inter-connected nodes, termed modules, represents genes with similar gene set membership in Liverome. To identify such modules, we define node similarity as the generalized topological overlap between nodes as introduced by Zhang and Horvath (2005), which is a measure based on number of shared neighbors in the network. More specifically, let a_{ij} be entries in the adjacency matrix A, and define the total connectivity of node i as:

$$k_i = \sum_j a_{ij}$$

The generalized topological overlap (GTOM) is then computed as:

$$GTOM(i,j) = \frac{\sum_u a_{iu}a_{uj} + a_{ij}}{\min(k_i, k_j) + 1 - a_{ij}}$$

And the associated distance metric is:

$$dissGTOM(i,j) = 1 - GTOM(i,j)$$

Nodes are then hierarchically clustered using dissGTOM as the distance metric and grouped into modules using the Dynamic Tree Cut method. Only modules with sizes are not smaller than 30 are selected for subsequent analysis. All module identification steps were carried out in R using the WGCNA package.

The identified modules were then searched for enrichment in known biological pathways represented in MSigDB, including BioCarta, Canonical pathways, GenMAPP, KEGG pathways, GO biological process and GO molecular function. Terms with less than 10 or greater than 500 members were excluded. Enrichment was computed using Fisher's exact test and all 6329 genes as background. Nominal p-values are adjusted using the Benjamini-Hochberg FDR to correct for multiple testing.

References

Langfelder P, Zhang B, Horvath S: **Defining clusters from a hierarchical cluster tree: the Dynamic Tree Cut package for R.** *Bioinformatics* 2008, **24:**719-220

Zhang B, Horvath S: **A general framework for weighted gene co-expression network analysis.** *Stat Appl Genet Mol Biol* 2005 **4:**Article17
